# In vitro hemo- and cytocompatibility of bacterial nanocelluose small diameter vascular grafts: Impact of fabrication and surface characteristics.

**DOI:** 10.1371/journal.pone.0235168

**Published:** 2020-06-24

**Authors:** Max Wacker, Viktoria Kießwetter, Ingo Slottosch, George Awad, Adnana Paunel-Görgülü, Sam Varghese, Maurice Klopfleisch, Dennis Kupitz, Dieter Klemm, Sandor Nietzsche, Katrin Petzold-Welcke, Friederike Kramer, Jens Wippermann, Priya Veluswamy, Maximilian Scherner

**Affiliations:** 1 Department of Cardiothoracic Surgery, University Hospital of Magdeburg, Magdeburg, Germany; 2 Department of Cardiothoracic Surgery, Heart Center of the University of Cologne, Cologne, Germany; 3 Department of Nuclear Medicine, University Hospital of Magdeburg, Magdeburg, Germany; 4 KKF Gesellschaft UG (haftungsbeschränkt), Jena, Germany; 5 Center for Electron Microscopy, University Hospital Jena, Jena, Germany; Michigan Technological University, UNITED STATES

## Abstract

**Objective:**

There is an increasing need for small diameter vascular grafts with superior host hemo- and cytocompatibilities, such as low activation of platelets and leukocytes. Therefore, we aimed to investigate whether the preparation of bacterial nanocellulose grafts with different inner surfaces has an impact on in vitro host cytocompatibility.

**Methods:**

We have synthesized five different grafts in a bioreactor, namely open interface surface (OIS), inverted (INV), partially air dried (PAD), surface formed in air contact (SAC) and standard (STD) that were characterized by a different surface roughness. The grafts (length 55 mm, inner diameter 5 mm) were attached to heparinized polyvinyl chloride tubes, loaded with human blood and rotated at 37°C for 4 hours. Then, blood was analyzed for frequencies of cellular fractions, oxidative products, soluble complement and thrombin factors. The results were compared to clinically approved grafts made of polyethylene terephthalate and expanded polytetrafluoroethylene. Additionally, blood platelets were labelled with ^111^Indium-oxine to visualize the distribution of adherent platelets in the loop by scintigraphy.

**Results:**

SAC nanocellulose grafts with the lowest surface roughness exhibited superior performance with <10% leukocyte and <50% thrombocyte loss in contrast to other grafts that exhibited >65% leukocyte and >90% thrombocyte loss. Of note, SAC nanocellulose grafts showed lowest radioactivity with scintigraphy analyses, indicating reduced platelet adhesion. Although the levels of reactive oxygen species and cell free DNA did not differ significantly, the levels of thrombin-antithrombin complexes were lowest in SAC grafts. However, all nanocellulose grafts exhibited enhanced complement activation.

**Conclusion:**

The systematic variation of the inner surfaces of BNC vascular grafts significantly improves biocompatibility. Especially, SAC grafts exhibited the lowest loss of platelets as well as leukocytes and additionally significantly diminished activation of the coagulation system. Further animal studies are needed to study in vivo biocompatibilities.

## Introduction

Despite the clinical success of large-diameter vascular grafts, synthetic grafts in small-diameter vessels (<6 mm) are rarely used because of poor patency rates, which limit their application in coronary and peripheral vascular bypass graft procedures [1, 2]. Several attempts have been made to test the application of tissue-engineered vascular grafts for the replacement of small-diameter vessels [3–5]. One promising concept is the use of the hydrogel bacterial nanocellulose (BNC) designed in tubular shape. Our first in vivo studies with implanted BNC grafts showed remarkable mechanical properties with excellent biocompatibility and we were able to improve the patency rate from 50% after three months to 80% after 9 months using a modified surface structure of the BNC grafts [6–8]. However, early occlusion in a significant number of cases led to the conclusion that the surface of the BNC grafts still does not possess sufficient anti-thrombogenic properties. Anti-thrombogenic properties remain pivotal during the initial phase after implantation until the graft is colonized by the recipient cells. Requirements for testing the anti-thrombogenic properties have been described recently and are given by the ISO 10993–4 (International Organization for Standardization) [9, 10]. We developed new techniques to modify the surface structure of BNC grafts. These modified grafts were characterized by differences in surface roughness. The aim of this study was to analyze the impact of different inner surfaces of BNC grafts produced by variation of the biotechnological procedure on the thrombogenic potential and blood biocompatibilities. An in vitro system was used to simulate the extracorporal blood circulation to get further insights into: a) the interaction between human blood cells and the newly modified grafts; b) the activation of the coagulation system; c) the activation status of the complement cascade system. The characteristics of these surface variated BNC grafts were directly compared to commercially available artificial small diameter grafts made of PET (polyethylene terephthalate) and ePTFE (expanded polytetrafluoroethylene).

## Materials and methods

### Design of bacterial nanocellulose grafts and surface characterization

**Fig 1:**
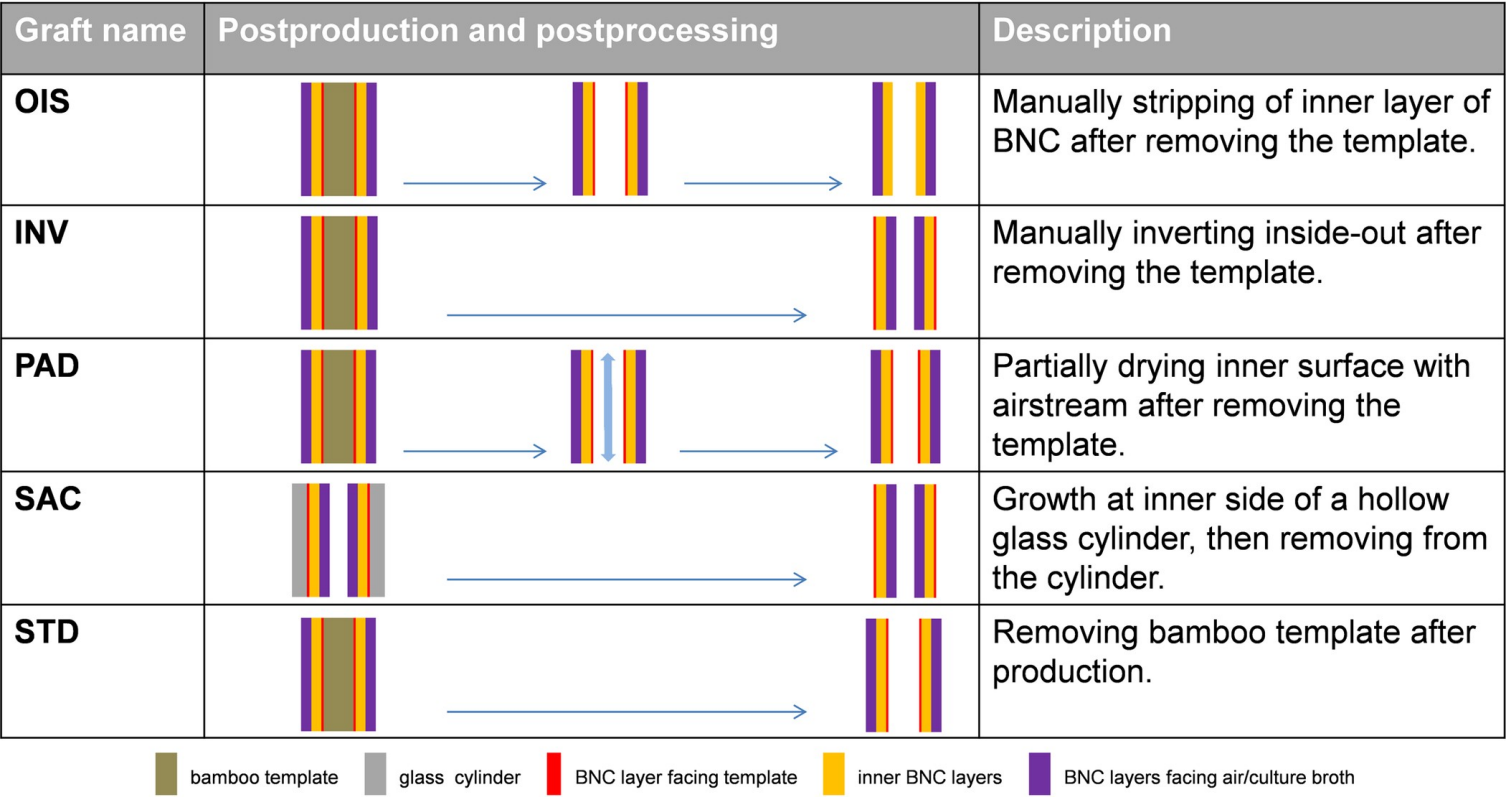
Graft production. Schematic representation of BNC grafts and their layered structure. Included are information regarding production procedure.

**Fig 2:**
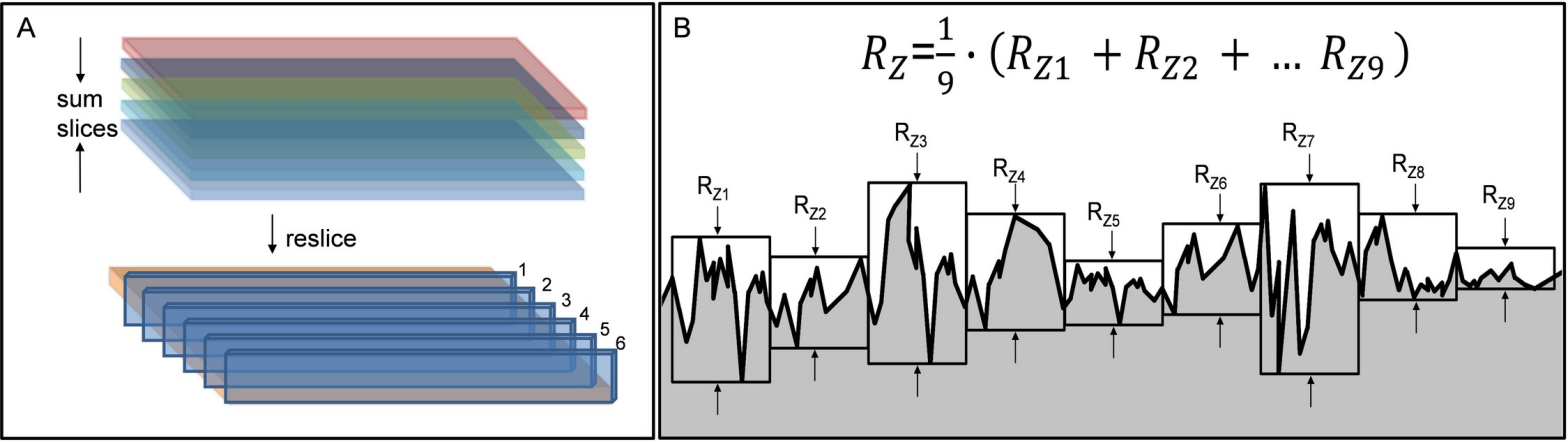
Determination of surface roughness. (A) Postprocessing of image stacks obtained by confocal laser scanning microscopy. (B) Measurement and calculation of surface roughness depth (*R*_*z*_) for each slice. $R_{\tilde{z}}$ represents the mean roughness depth for all slices.

The bacterial nanocellulose grafts were produced by Mobile Matrix Reservoir Technology by KKF Gesellschaft UG (haftungsbeschränkt) in Jena as described and patented earlier [11–13]. Briefly, cylindrical templates are moved periodically between air space and a reservoir, which is filled with liquid culture medium and bacteria of the genus *Komagataeibacter xylinus* (German Collection of Microorganisms and Cell Cultures GmbH, DSM 32384). During dipping, the template is loaded with culture medium and bacteria. After leaving the liquid, the BNC formation takes place exclusively on the template surface. To achieve surface variations of the grafts, we adapted different production techniques using two different templates (bamboo and glass) as described in Fig 1. Five different inner surface structures of BNC grafts were generated, namely i) open interface surface (OIS); ii) inverted (INV), iii) partially air dried (PAD), iv) surface formed in air contact (SAC) and (v) standard (STD). Bamboo templates were used for all BNC grafts, except for SAC, as bamboo can easily be wettened by the culture broth, avoiding the culture broth to drip off from the template during the production process. Since bamboo templates always possess a certain fibre structure that is giving an imprint to BNC graft, we differently produced grafts with a hollow, cylindrical glass template in a way that the luminal surface of the BNC graft was not in contact with the glass template as shown in Fig 1. Of note, all the produced BNC grafts showed uniformity in length (50 mm), diameter (5.0 mm) and wall thickness (1.0 mm). The luminal side of each modified BNC graft was characterized using scanning electron microscopy (SEM) and by confocal laser scanning microscopy (CLSM). The sample preparation for SEM was performed as described previously [12]. The image stacks obtained from CLSM were resliced and *R*_*z*_ value (μm) was measured for each slide separately using Fiji 64-bit for Windows [14] and Image-pro plus (Version 6.0.0.260, Media Cybernatics, Rockville, USA) in order to determine the mean surface roughness depth, indicated by $R_{\tilde{z}}$ (Fig 2).

### Chandler loop assembly

**Fig 3:**
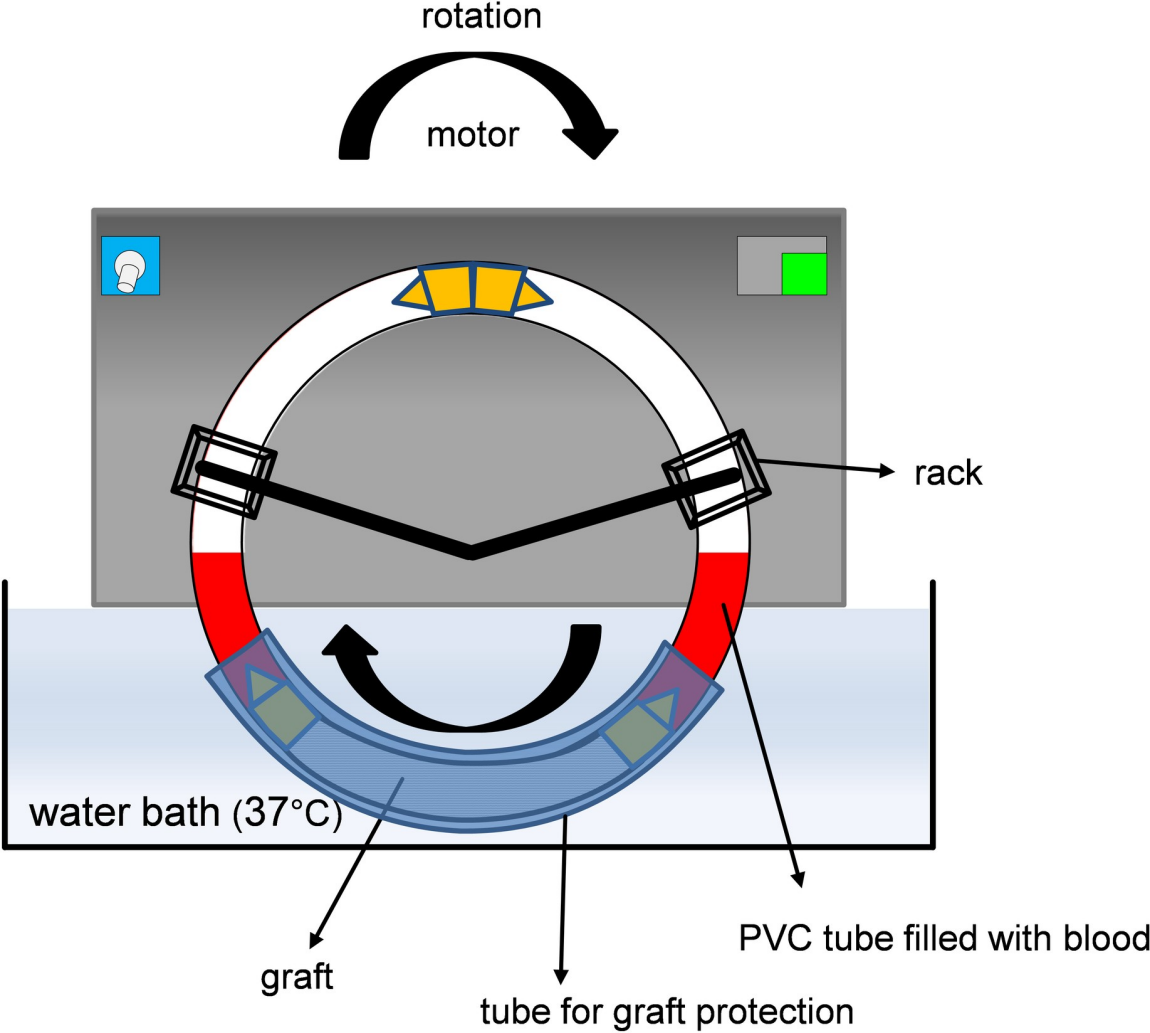
Assembly of the Chandler loop system. To protect the graft material and in order to prevent osmosis, the graft section of the loop was covered with a bigger tube and sealed with tape.

To characterize the interaction between different surface variated BNC grafts with human blood cells while simulating a continuous blood flow, we used a modified Chandler loop model as described by Fink et al. [5]. Briefly, polyvinyl chloride (PVC) tubes with an inner diameter of 5.0 mm and a wall thickness of 1.0 mm (VWR #228–1752) were fixed to metal connectors to form loops. For the experimental setup, PVC tubes with a total length of 450 mm were attached to 50 mm long grafts (BNC, PET or ePTFE) with metal connectors that were made of thin wall stainless steel (Sawade Edelstahlrohre, Gottmadingen, Germany) with an inner diameter of 5.0 mm. Loops with a 50 mm long PVC tube alone served as controls. All parts of the loops (PVC tubes, metal connectors) were heparinized using a lab site heparin coating kit (Corline Systems AB, Uppsala, Sweden) according to manufacturer’s recommendations, giving a final concentration of 0.9 μg heparin/cm^2^. The loops were then secured on a customized rack with a motor unit and rotation speed control and rotated in a water bath as shown in Fig 3. The Chandler loop system was obtained from Ebo Kunze Industriedesign, Neuffen, Germany.

### Human subjects, blood sample collection and processing

In total, this study recruited 21 healthy and medication-free volunteers from the department coworkers. Approval was obtained from the ethics committee of the University of Magdeburg (file number 88/18). For each experiment, 50 ml of fresh blood was collected in a beaker glass containing unfractionated heparin giving a final concentration of 1.5 IE/ml (Rotexmedica, Trittau, Germany). The loops were immediately filled with 5.0 ml of the heparinized blood leaving air space and rotated in a water bath at 37°C at 30 rpm for 2 hours and 4 hours. For each measurement, the test loop with attached graft was run together with a control loop consisting of heparinized PVC alone. Here, we determined the efficient rotation time suitable for the conduction of further experiments and therefore compared the frequencies of thrombocytes and leukocytes after rotation for 2 and 4 hours (n = 3). We did not observe any significant changes occurring after 2 hours, but significant leukocyte and thrombocyte frequency changes occurred at 4 hours (see results). Thus, the rotation time of 4 hours was selected for the experiments. At the end of rotation, 1.0 ml of blood was immediately taken from the loops for measuring reactive oxygen species (ROS). For plasma collection, blood was centrifuged at 3500×g for 20 minutes and the plasma samples were aliquoted and stored at -80°C for further analyses. The remaining blood was collected in a polypropylene tube containing K3 Ethylenediaminetetraacetic acid (EDTA) to give a final concentration of 1.6 mg/ml and stored on ice for further analysis (Sarstedt Tube 5 ml, 75x13 mm, K3E, Sarstedt AG & Co, Nümbrecht, Germany). Moreover, 5.0 ml of heparinized blood in a beaker glass was kept at room temperature without agitation for 4 hours. These samples served as controls to ensure proper heparinization. Prior to start of the Chandler loop experiments, 5.0 ml of blood was immediately used to quantify ROS levels and to determine baseline levels of analytes in plasma.

### Frequencies of blood cells

A conventional blood cell frequencies analysis from EDTA-blood was made in the Institute for Clinical Chemistry and Pathobiochemistry, Otto-von-Guericke University Magdeburg, on the day of the experiment to determine the frequencies of leukocytes, erythrocytes and thrombocytes in the blood by flow cytometry (Sysmex XE-5000, Sysmex Europe GmbH, Norderstedt, Germany).

### Reactive Oxygens Species (ROS) measurements

The ROS levels were measured in human fresh blood as described by Golightly [15]. 100 μl of blood samples were supplemented with 20 μl of dihydrorhodamine 123 (Sigma #D1054) and 900 μl bovine serum albumin. The samples were incubated for 15 minutes in a water bath with shaker at 37°C and washed with phosphate buffered saline solution (PBS). The samples were then centrifuged at 1500 rpm for 3 minutes and the supernatant was discarded. The erythrocytes were lysed with ammonium chloride, followed by centrifugation, washing and resuspension of the pellet in PBS. The fluorescent intensity of rhodamine 123 was measured at 485 nm and 535 nm in a microplate reader (Synergy HT, BioTek Instruments, USA) and analyzed with Gen5 (Version 1.11.5, BioTek Instruments, USA). All samples were run in triplicates.

### Measurement of cell-free DNA (cfDNA)

The plasma levels of cfDNA were quantified by Quant-iT Pico Green dsDNA assay, according to the manufacturer’s instructions (Invitrogen GmbH, Darmstadt, Germany). The fluorescence intensity was measured at excitation and emission wavelengths of 485 nm and 530 nm in a microplate reader (Synergy HT, BioTek Instruments, USA). A defined amount (ranging 0 to 2 μg/ml) of calf thymus DNA (Sigma Aldrich) was used for the standard calibration curve; only the linear range of the calibration curve was used. All samples were run in duplicates.

### Thrombin-Antithrombin complex (TAT) and complement components measurements

Circulating plasma levels of TAT and complement factors such as terminal complement complex (TCC) and anaphylatoxin (C3a) were quantified using commercially available enzyme-linked immunosorbent assay (ELISA) kits according to manufacturers’ instructions (TAT: Catalogue No #108907; Abcam plc, Cambridge, UK; TCC: Catalogue No #HK328-02; Hycult Biotech, Uden, NL; C3a: Catalogue No #BMS2089; Invitrogen GmbH, Darmstadt, Germany). All samples were run in duplicates.

### Cryosectioning and immunofluorescence

After a 4 hour Chandler loop rotation, blood was drawn out of the loops and the loops were flushed with 30 ml of PBS to remove excessive cells which were not firmly adhering to the grafts surfaces. BNC grafts were cut and embedded in Tissue-Tek O.C.T. compound (Sakura Finetek Europe B.V., Alphen aan den Rijn, NL) and stored at -80°C. After freezing, cryosectioning was performed using a Leica CM 1950 cryostat (Leica Biosystems, Nussloch, GER). Here, activated thrombocytes were detected using rabbit anti-human CD62P (Biorbyt #orb416329) as primary antibody and respective donkey anti-rabbit Alexa Fluro 488 (Jackson ImmunoResearch #711-547-003) as secondary antibody. Likewise, erythrocytes were detected using mouse anti-human CD235a (Biorbyt #orb248903) as primary antibody and respective donkey anti-mouse IgG Cy3 (Jackson ImmunoResearch #715-167-003) as secondary antibody. Upon acetone fixation, the graft slices were blocked with 3% standard donkey serum and then incubated with the primary antibodies at the dilution of 1:50 in 3% donkey serum overnight. After washing, the fluorescence dye conjugated secondary antibody at the dilution of 1:500 in 3% donkey serum was incubated on the slice in the dark for one hour. The slices were then counterstained with DAPI (Sigma D9542) to stain the nuclei before mounting and microscopy (EVOS Auto 2, ThermoFisher Scientific, Massachusetts, USA).

### Detection of adherent thrombocytes by Indium-111 oxine radioactive labelling

To further characterize the distribution of adhering thrombocytes on the luminal BNC surface and to rule out possible bias due to pseudothrombocytopenia as a result of thrombocyte clumps, thromobcytes were labelled with radioactive indium-111 oxine as describe by Rodrigues et al. and rotated in the Chandler loops after resuspension in the other blood components [16]. By the end of experiment, blood was collected and centrifuged to produce platelet rich plasma. Acid-citrate-dextrose-A (C3821) was used as anticoagulant (0.2 ml/ml blood). Purity of the isolated thrombocytes was confirmed by flow cytometry (Sysmex XE-5000, Sysmex Europe GmbH, Norderstedt, Germany). From 60 ml of blood, a total number of 1288x10^6^ thrombocytes was isolated and diluted in 4 ml of plasma. A total of 37.37 megabecquerels (MBq) ^111^In-oxine was added to the cell suspension and incubated for 10 minutes. After labelling, the thrombocytes and remaining plasma were resuspended into the remaining blood components to a final volume of 55 ml. Then, the loops were filled with 5 ml of blood each and rotated in a water bath for 4 hours as described above. Two loops with heparinized PVC were used as controls, one was rotating inside the water bath, the other one was kept outside the water bath at room temperature without any agitation. After rotation, the loops were emptied and flushed with 10 ml of PBS. To determine the radioactivity and visualize the thrombocyte distribution on the graft surface, a scintigraphy was conducted with a gamma camera (MiE Syngula Scintron, MiE medical imaging electronics GmbH, 23845 Seth, Germany) for each loop. The exposure time was set to five minutes. As a reference for later quantitative analyses, we used a scinitgraphy of a point source with known radioactivity (5.313 MBq). All acquired images were analyzed using Fiji 64-bit for Windows [14]. Given that each emitted photon striking the gamma camera sensor increases the corresponding pixel by one, each pixel shows a designated value correlating positively with the amount of radioactive labelled thrombocytes adhering to the loop. These values were used to calibrate and colorize the pictures. A region of interest (ROI) was defined in order to allow for comparison between the grafts. The RawIntDent (raw integrated density: defined by the sum of the values of the pixels in the ROI) was measured for the image of each loop and also for the point source that served as a reference.

### Statistics

Data were analyzed using Prism 8 (GraphPad Software, San Diego, USA). Kruskal-Wallis Test and Dunn’s multiple comparison test were conducted for all experiments. The level of significance was set to p<0.05.

## Results

### SAC BNC graft possess diminished surface roughness

**Fig 4:**
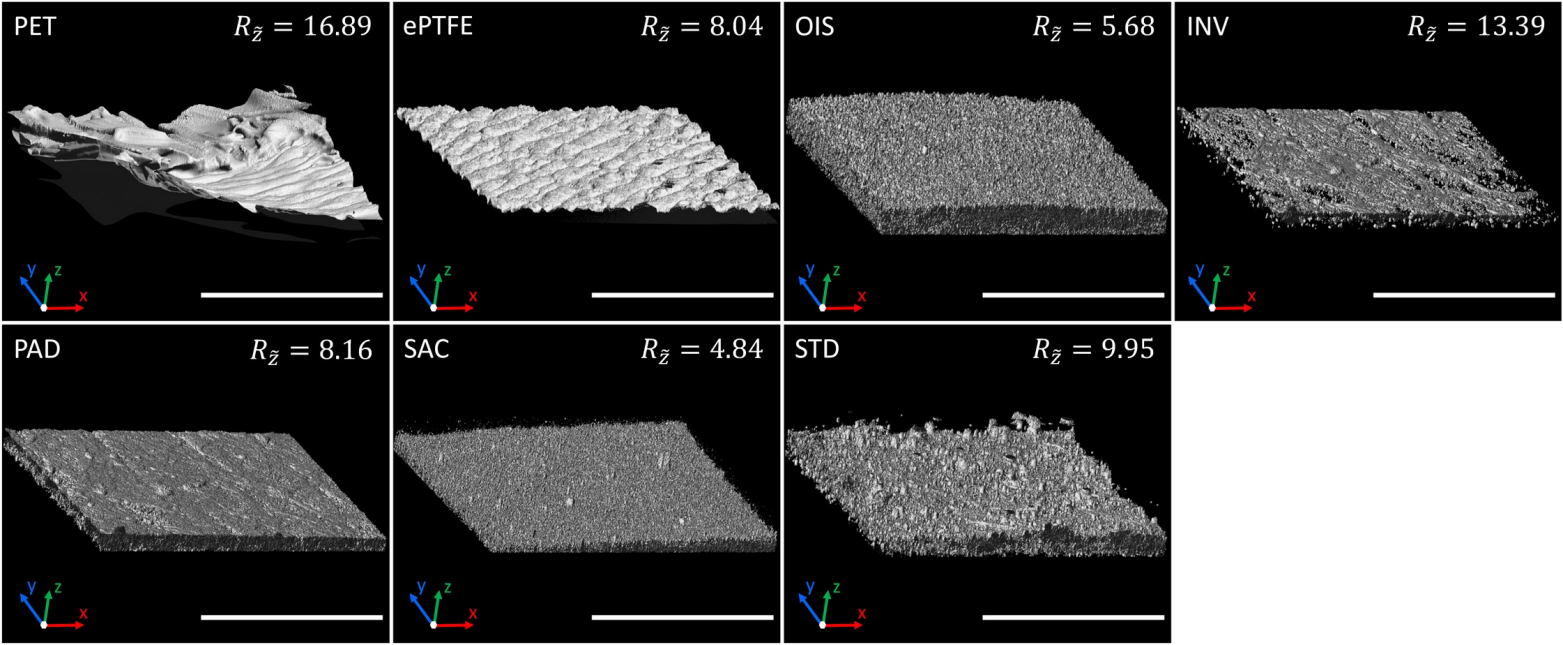
Confocal microscopy. The luminal surface of different types of described graft materials in this study was characterized by confocal laser scanning microscopy. The mean roughness depth $R_{\tilde{z}}$ (μm) is given for each material. The scalebar represents 200 μm.

**Fig 5:**
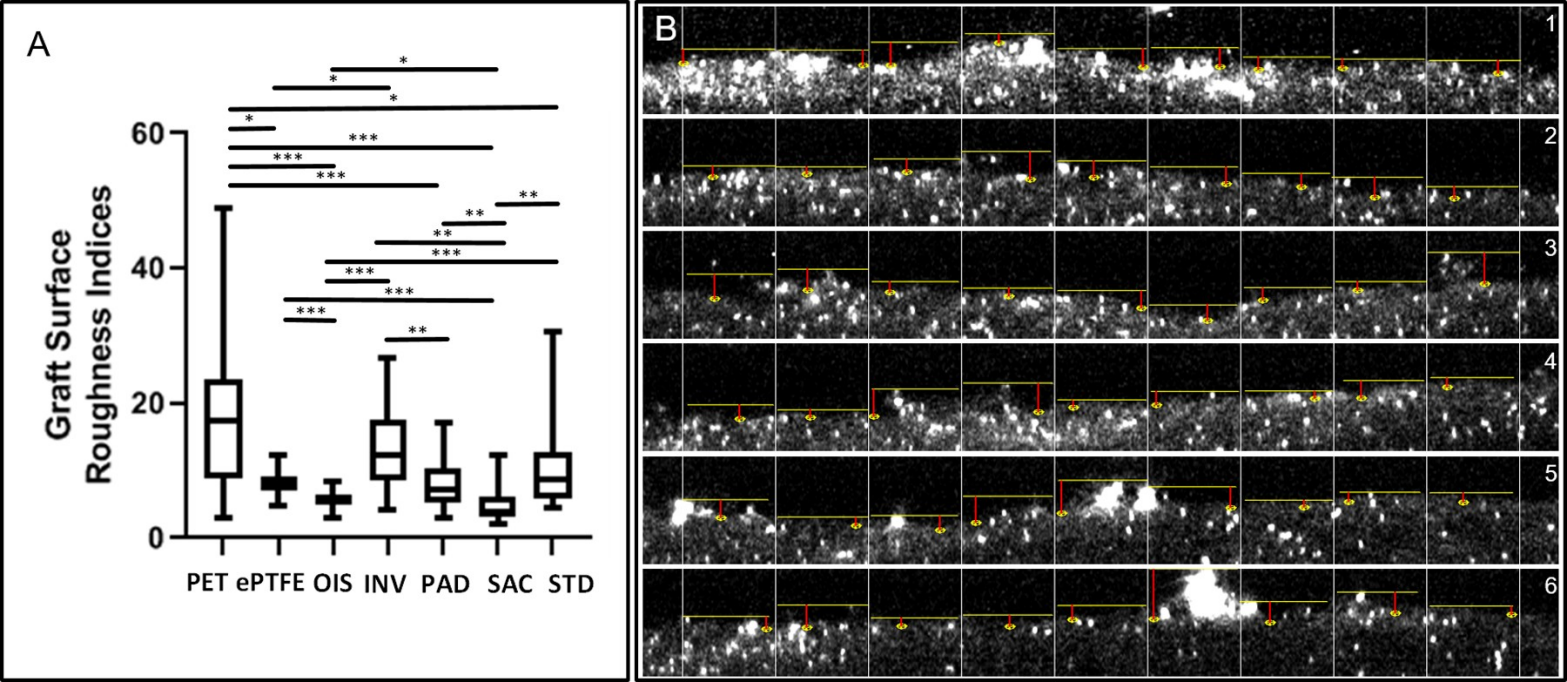
Graft surface roughness and representative measurements. A) The box plots show the differences of the mean roughness indices ($R_{\tilde{z}}$, μm) between the different graft surfaces. The statistical significances between the different grafts were as follows: p<0.0001 for PET versus OIS, PAD and SAC; ePTFE versus OIS and SAC; OIS versus INV and STD; p = 0.0002 for INV versus SAC; PAD versus SAC, SAC versus STD and INV versus PAD, p = 0.0028 for PET versus ePTFE, p = 0.0173 for PET versus STD; p = 0.0292 for ePTFE versus INV and p = 0.0156 for OIS versus PAD. The lower, mid and upper horizontal lines of the boxes represent 25^th^, 50^th^ and 75^th^ percentiles, respectively; the vertical lines extend from the 10^th^ to the 90^th^ percentile. B) Image shows the calculation for $R_{\tilde{z}}$ on the resliced CLSM stack for STD graft. The red line indicates the surface depth measured for each sub-division of the image. *p<0.05, **p<0.01, ***p<0.0001.

**Fig 6:**
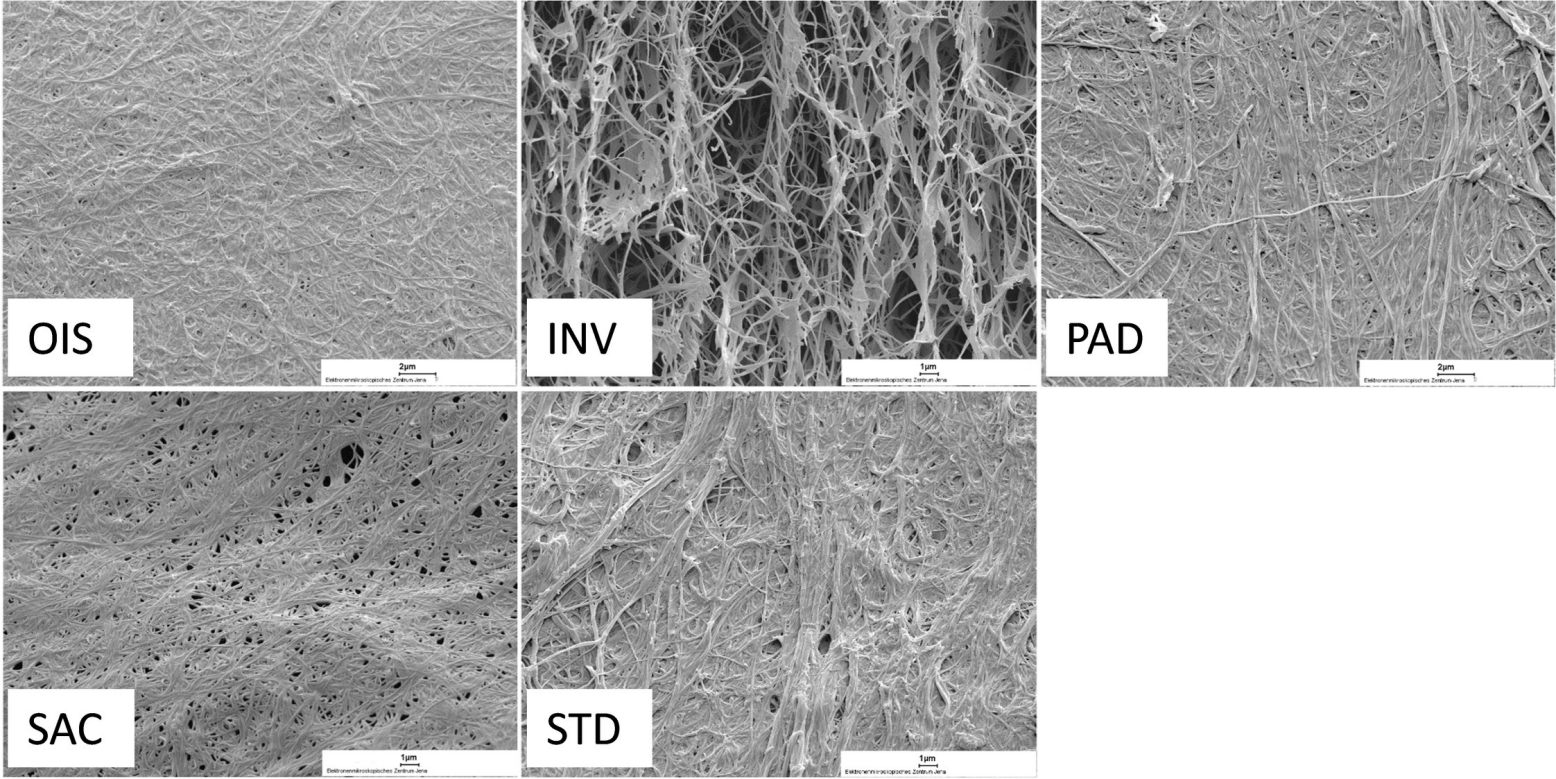
Scanning electron microscopy. SEM images of the different BNC grafts showing the luminal surface and the fiber structure of the freeze dried graft (magnification 4000x).

Upon surface characterization of modified BNC grafts by CLSM, we observed that the SAC grafts exhibited the lowest surface roughness followed by OIS grafts, as indicated by mean roughness index $R_{\tilde{z}}$ in Fig 4 and also reaching the level of statistical significance when compared to the other BNC grafts and the commercial grafts (Fig 5). The fibre structure of the BNC surfaces is displayed in the SEM images, where SAC and comparatively OIS are less fibrous (Fig 6).

### SAC BNC grafts show decreased loss of circulating blood thrombocytes and leukocytes

**Fig 7:**
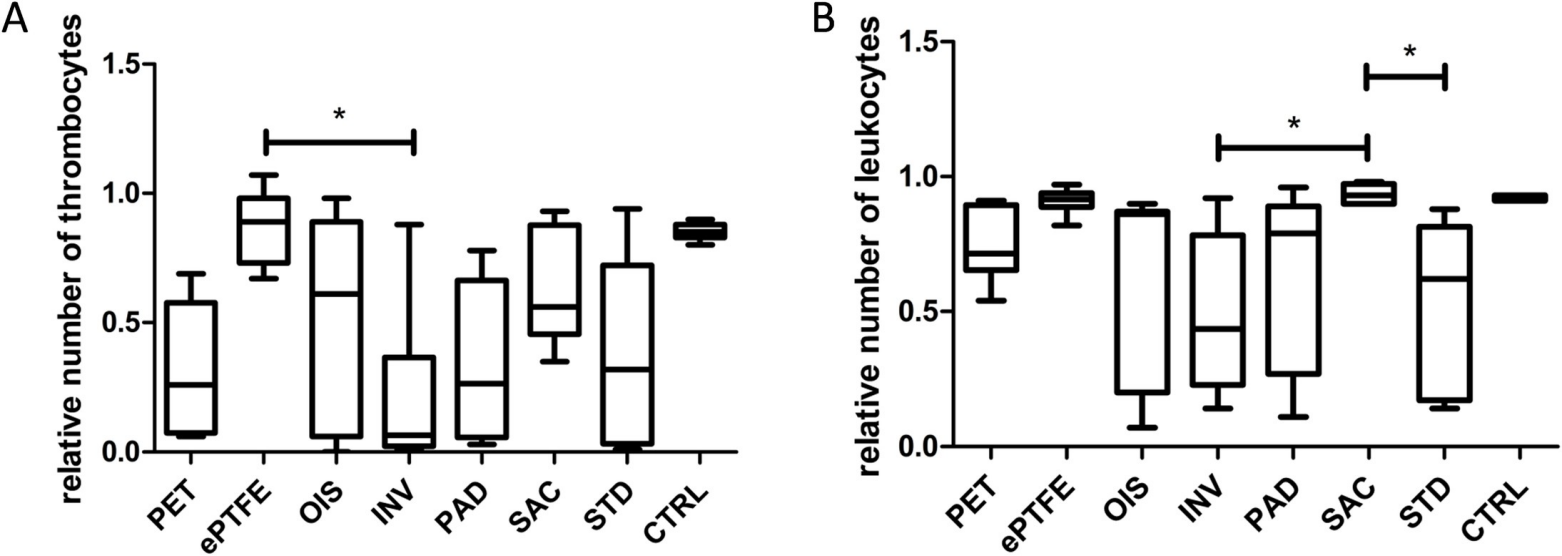
Blood cell count. (A) Frequencies of blood circulating thrombocytes. (B) Frequencies of blood circulating leukocytes. The values represented are relative to the baseline cell frequencies that were measured immediately upon blood drawing. N = 6 (PET, ePTFE, SAC), n = 7 (OIS), n = 8 (INV, PAD, STD), n = 56 (CTRL). The lower, mid and upper horizontal lines of the boxes represent 25^th^, 50^th^ and 75^th^ percentiles, respectively; the vertical lines extend from the 10^th^ to the 90^th^ percentile. *p<0.05.

Strikingly, the level of circulating thrombocytes did not decrease in SAC BNC tubes in comparison to other BNC grafts, however, these differences did not reach statistical significance (Fig 7).The frequency of thrombocytes in ePTFE grafts was almost comparable to the control whereas we noticed significantly reduced blood thrombocytes in inverted BNC grafts when compared to ePTFE grafts (p = 0.0155). Further, SAC BNC grafts, ePTFE and control showed similar results with regard to leukocyte consumption. We observed significantly decreased frequencies of leukocytes in both inverted (p = 0.0210) as well as standard BNC grafts (p = 0.0215) when compared to the SAC BNC graft. Red blood cells did not exhibit a reduction greater than 10% for any of the commercial (PET and ePTFE) or BNC grafts. Further leukocyte analysis, with regard to the subtypes neutrophils, monocytes and lymphocytes, did not show significant changes between the grafts. However, there was a trend for decreased frequencies of circulating blood monocytes for both standard and inverted BNCs, whereas the latter exhibited a trend for decreased frequencies of neutrophil compared to other grafts (S1 Table).

### SAC BNC grafts exhibit a reduced deposition of activated thrombocytes and leukocytes

**Fig 8:**
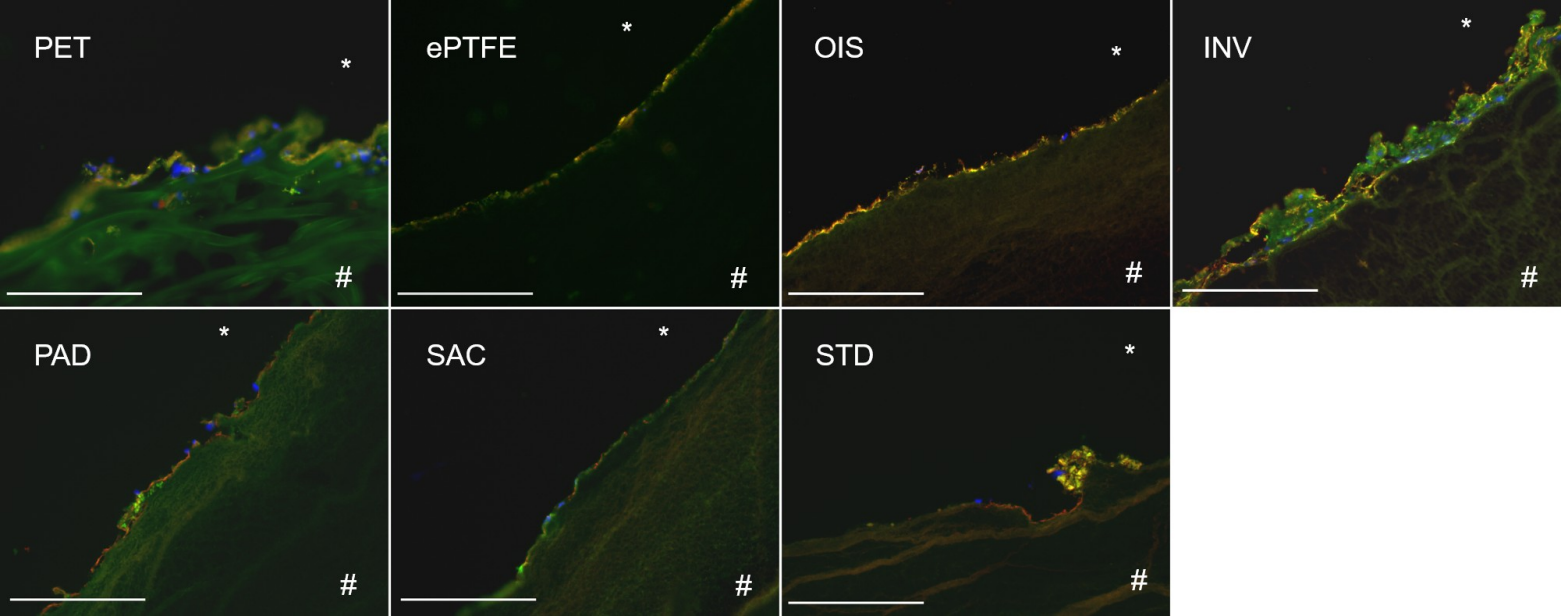
Immunofluorescence staining of different grafts succeeding 4 hours of rotation in the Chandler loop system. Staining against CD62P for activated thrombocytes (green), CD235a for red blood cells (red) and 4′,6-diamidino-2-phenylindole (DAPI) for nucleated cells (leukocytes, blue) on the luminal side of the graft materials. Scale bar represents 200μm.

When cryosectioned BNC grafts were fluorescently stained for activated thrombocytes, leukocytes and red blood cell markers, we observed that the SAC BNC grafts exhibited a reduced cell adhesion capability and therefore the adherence of activated platelets, leukocytes or red blood cells were found minimal when compared to STD BNC grafts (Fig 8). Of note, these minimally found cells on SAC BNC graft did not show any visible signs of aggregation or clotting (thrombi formation) in comparison to the STD BNC grafts that showed distinct and noticeable aggregates containing thrombocytes and leukocytes adhering to the BNC surface. Apart, both INV and PET grafts showed increased cell adhering capacity with thrombocyte and leukocyte deposition and PAD to a lesser extent. Further, the ePTFE and OIS grafts were almost comparable to the SAC BNC graft with no obvious thrombocyte aggregates on the luminal side.

### SAC BNC grafts exhibit lowered prothrombogenic potential

**Fig 9:**
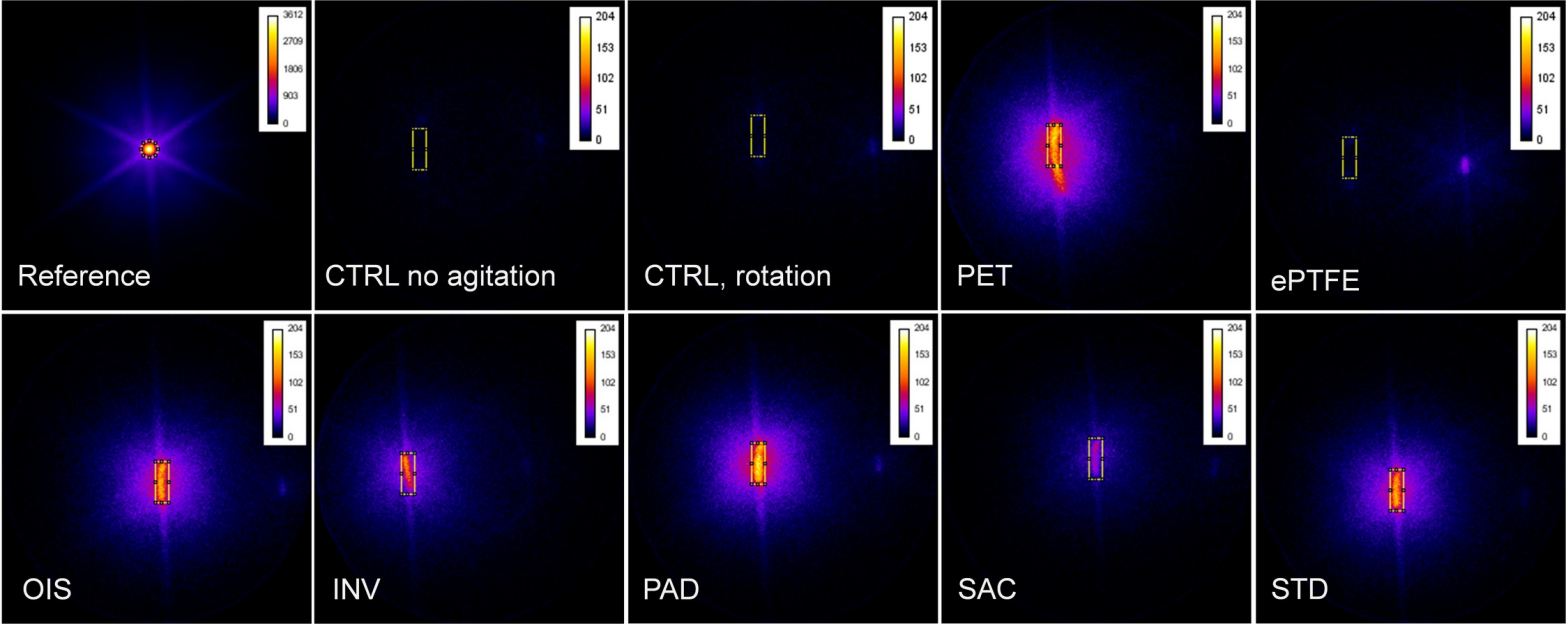
111In-oxine labelling of thrombocytes. Colored scintigraphies of the loops, with known amount of 111In-oxine labelled thrombocytes in whole blood, between different types of grafts succeeding 4 hours in a Chandler loop system. The yellow rectangle represents the region of interest for measurements of pixel values. The blue glow around the yellow/red shining BNC with adherent thrombocytes is an artifact. The color scale was set from black (no radioactivity) to white (high radioactivity) and a point source, 5.313 MBq, served as a reference. (CTRL: Control).

Interestingly and in accordance with our immunofluorescence staining results, we found that the SAC BNC grafts showed the lowered radioactivity, indicating the decreased number of thrombocytes adhering to the material (Fig 9). The radioactivity of the SAC BNC graft was almost comparable to ePTFE, showing lowest thrombocyte adherence to the surface compared to all the studied grafts. Further, we observed that the thrombogenic ability of the OIS graft was more comparable to STD, PAD and PET grafts. In particular, the number of adherent thrombocytes in SAC BNC graft was approximately twice as high than the ePTFE grafts, whereas all the other grafts (except SAC) exhibited six times higher thrombocyte adherence when compared to the ePTFE grafts. However, there were nearly no thrombocytes adhering to the control heparinized PVC loops under both dynamic and static conditions. The cell frequencies were not determined in the blood after ^111^In-oxine labelling due to radioactive-based regulatory restrictions. However, by knowing the number of thrombocytes before labelling with ^111^In-oxine and by measuring the radioactivity of the point source as a reference, the radioactivity and respective count of adherent thrombocytes was calculated using common three sets calculations (S2 Table).

### SAC BNC grafts possess lesser impact in activating the coagulation system

**Fig 10:**
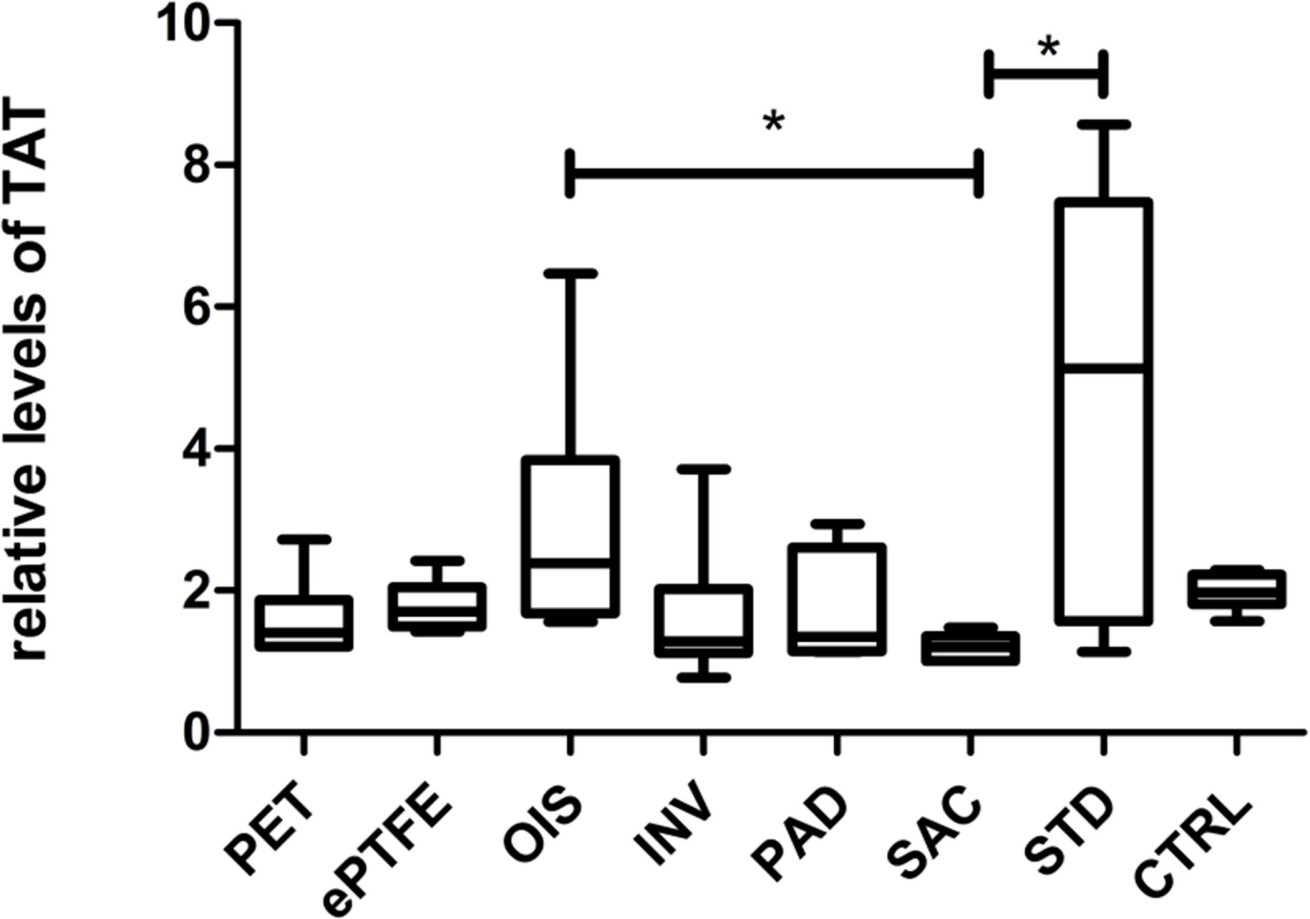
Plasma levels of Thrombin-antithrombin (TAT) complex. The values represented are relative to the baseline TAT levels immediately upon blood drawing. N = 6 (PET, ePTFE, INV, PAD, SAC), n = 7 (OIS, STD), n = 44 (CTRL). *p<0.05.

SAC BNC grafts showed the lowest TAT generation in comparison to other BNC grafts and commercially available grafts as shown in Fig 10. The plasma TAT levels for SAC BNC grafts were significantly reduced when compared to STD BNC (p = 0.0323) and interestingly to OIS BNCs grafts (p = 0.0286) (Fig 10). All the derived numerical values with standard deviations are summarized (S3 Table).

### SAC BNC graft do not exhibit favourable impact on the complement systems

**Fig 11:**
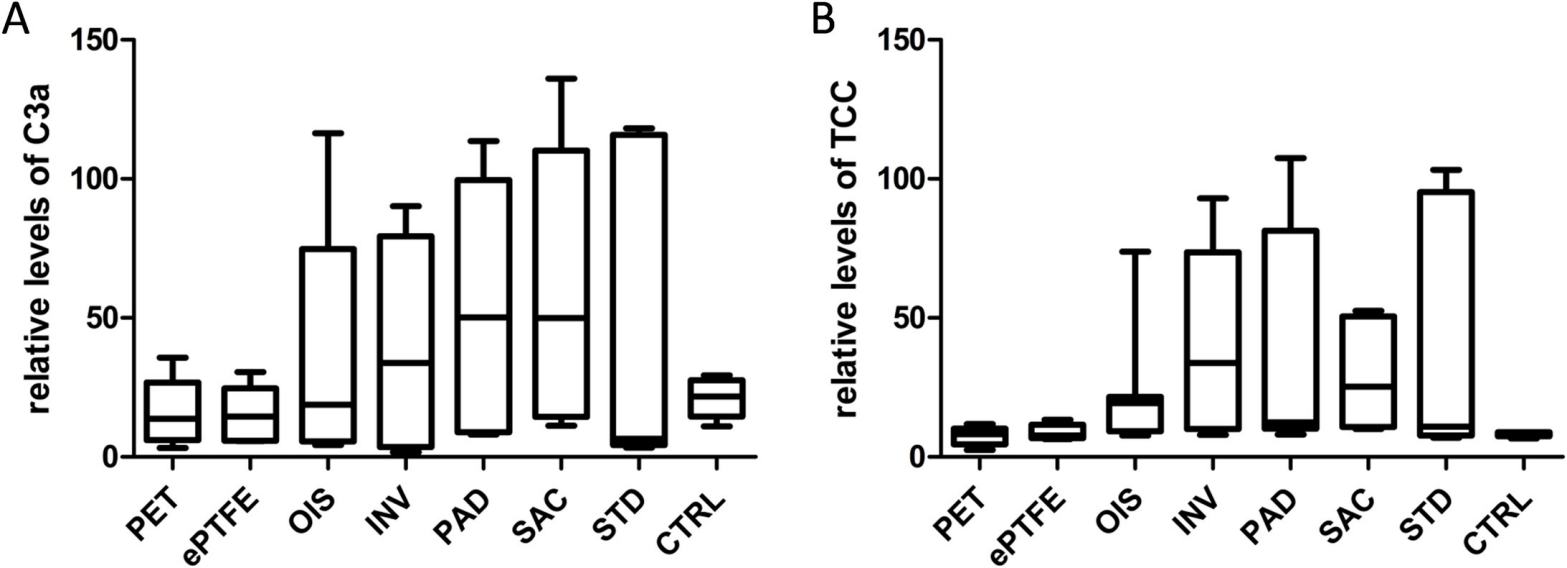
Activation of the complement system. (A) Plasma levels of C3a. (B) Plasma levels of TCC. The values represented are relative to the baseline C3a and TCC levels immediately upon blood drawing. N = 6 (PET, ePTFE, INV, PAD, SAC), n = 7 (OIS, STD), n = 44 (CTRL).

All the analyzed BNC grafts showed increased plasma levels of C3a, with SAC BNC exhibiting the highest of all other grafts, although not reaching the level of statistical significance between the grafts (Fig 11). But, evidently, the plasma levels of C3a for commercially available grafts (PET and ePTFE) remained low. Furthermore, all the BNC grafts displayed increased plasma levels of TCC, with SAC and OIS BNC grafts being reduced and demonstrating no obvious statistical differences between the grafts. Increased variations were observed in all the analyzed BNC grafts for plasma C3a and TCC levels. All the derived numerical values with standard deviations are summarized in S3 Table.

### SAC BNC grafts did neither support the reduction of oxidative system nor the circulating extracellular DNA

**Fig 12:**
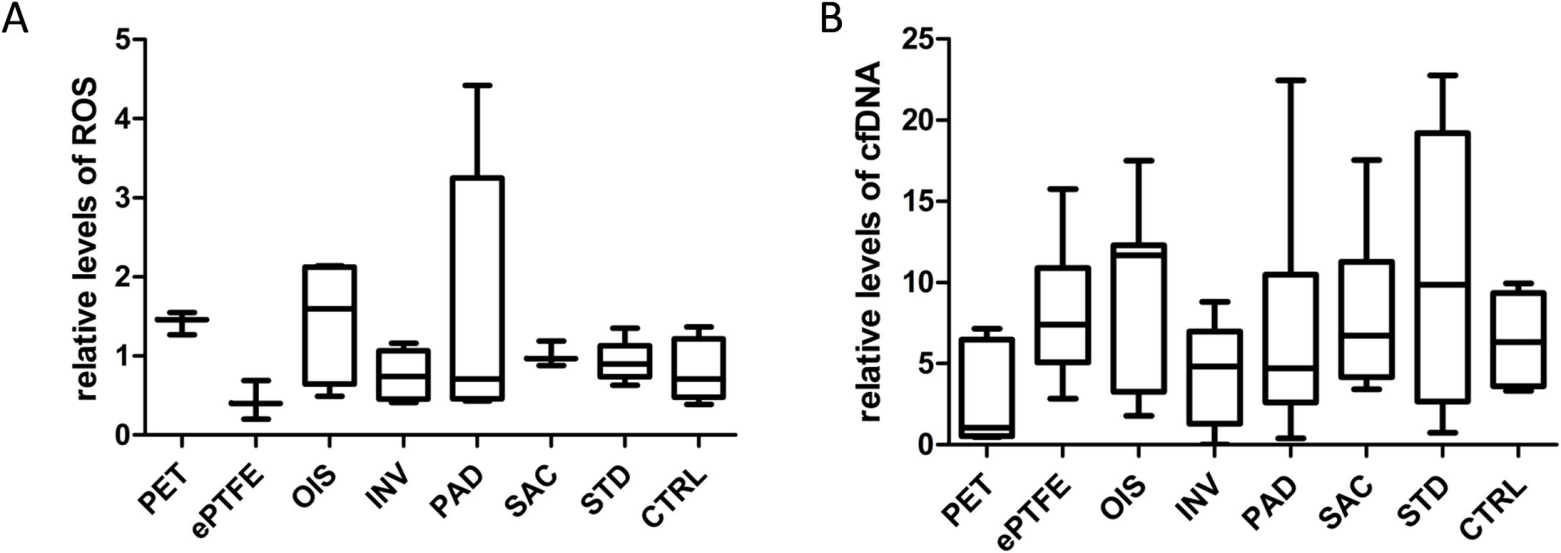
ROS formation and release of cfDNA. (A) Plasma levels of ROS. n = 3 (PET, ePTFE, SAC), n = 4 (OIS), n = 5 (INV, PAD, STD), n = 28 (CRTL). (B) Plasma levels of cfDNA. m = 6 (PET, ePTFE, SAC), n = 7 (OIS), n = 8 (INV, PAD, STD), n = 49 (CTRL). The values are represented as relative to the baseline levels immediately upon blood drawing.

Overall, there were no significant differences amongst the analyzed grafts (Fig 12). However, we observed that PAD and OIS BNC grafts exhibited 1.5 fold increase in the levels of blood ROS when compared to other grafts. Concerning plasma cfDNA levels, we observed no significant differences between the grafts. All the derived numerical values with standard deviations are summarized (S3 Table).

## Discussion

Our study remains the first to investigate the cytocompatibility of five different surface variated BNC grafts in a Chandler loop system, which is an artificial circulatory platform as recommended by the DIN ISO 10993–4. The Chandler loop system is less traumatic to the blood used and exhibits laminar blood flow in contrast to pump-driven systems. Therefore, we chose this system to analyze the blood biocompatibility of BNC grafts. Since platelets and leukocytes (majorly monocytes, neutrophils and T cells) were well reported to be involved in thrombosis and inflammation and thereby leading to occlusion of the implanted graft [17], we mainly focused on these blood cell populations.

Interestingly, in our study, the circulating blood thrombocytes and leukocytes in SAC BNC grafts were not curtailed and were almost comparable to commercial ePTFE grafts and control loops, whereas other BNC grafts provoked reduction in thrombocyte and leukocyte numbers. This is attributed to the increased adherent capability of circulating thrombocytes and leukocytes to the blood contacting surface of the analyzed grafts that differed in their surface roughness properties. Here, it was experimentally confirmed that the surface roughness positively correlates with the number of adherend platelets, where commercial PET grafts with increased surface roughness ($R_{\tilde{z}}$ = 16.89) tend to possess the highest number of thrombocytes that were attached to the surface (40799x10³) in contrast to the SAC BNC grafts with decreased surface roughness ($R_{\tilde{z}}$ = 4.84) that comparatively supported lesser platelet attachment (13936x10³). Several studies have reinforced this concept where blood compatibility of the grafts with respect to platelet adherence and subsequent activation of coagulation cascade are dependent on the fiber diameter and the surface roughness [18–21]. Very interestingly, in comparison to the SAC BNC grafts, the commercial ePTFE grafts displayed the lowest number of adhering thrombocytes, while they did not show the lowest surface roughness ($R_{\tilde{z}}$ = 8.04). This might be explained by different protein adsorption characteristics of BNC compared to ePTFE. It is generally accepted that hydrophobic surfaces, such as ePTFE, show a higher protein adsorption compared to hydrophilic surfaces, such as BNC [22]. However, it has already been shown that ePTFE exhibits high resistance to protein adsorption compared to PET, even upon 48 hours of incubation [23]. Besides, the nanometer dimensions of BNC fibres, the structural elements, result in a larger surface area thereby facilitating the strong interaction between cellulosic moieties with surrounding compounds or proteins [24]. Due to the fibre structure and the porosity of BNC surface, it might therefore be possible that the BNC grafts exhibited distinct protein adsorption properties, despite their hydrophilic nature, which needs further investigation. While protein adsorption is a complex process with many variables, it has already been reported that bacterial cellulose is capable of adsorbing proteins [25, 26]. During such protein-graft surface interactions, the cell adhesive arginine-glycine-aspartic acid (RGD) peptide-containing adhesion protein (Vitronectin, like fibronectin) presented in circulating plasma is responsible for increased anchoring of thrombocytes and leukocyte on BNC grafts, while the circulating plasma immunoglobulins (IgG) adhering to the BNC grafts might activate the platelets through their cell membrane bound Fc gamma receptors (FcγRs) [27–29]. Thus, tailoring the surface chemical composition and the functional groups with the surface roughness and topography determines the magnitude of protein adsorbing attributes of a graft. The kinetics of plasma protein adsorption on surface modified BNC grafts and their surface directed control of adsorbed protein in manipulating the thrombocyte and leukocyte adhesion mechanism and function remains to be an important issue for further investigation and clarification.

Since the surface roughness of the BNC graft was reported to be similar to the luminal surface of human veins [21], we assumed the conventional way of platelet aggregation and activation on the STD BNC graft ($R_{\tilde{z}}$ = 9.95 as roughness index). This was confirmed by demonstrating an increased platelet adhesive and activation, including leukocytes and activation of the coagulation system. Since TAT complex is considered as a surrogate marker for thrombin formation, we found that the SAC BNC grafts exhibited decreased generation of TAT, indicating a correlation of TAT complex generation to the blood activation as well as to the roughness of the grafts [18]. On the other hand, we noticed that the OIS BNC grafts are similar to SAC BNC graft with respect to surface roughness, but nevertheless they exhibited significantly higher TAT generation when compared to SAC. Few reports have stated that grafts with smoother surface could tend to activate the coagulation cascade, and the adsorption capacity of plasmatic proteins seems to be a key factor for activation of the coagulation system, probably outweighing the benefits of decreased surface roughness [30]. One, albeit hypothetical explanation might be that the removal of the inner fibre layer of the OIS BNC grafts could lead to disunited fibre ends. In contrast to SAC grafts, these fibre ends are not integrated into the graft surface, but exposed to their surrounding. The crystalline structure of the BNC fibre network will be destroyed by tearing layers apart, leading to an increased degree of molecule disorder by exposing the amorphous region with accessible hydroxyl groups. These accessible hydroxyl groups could lead to a higher amount of protein adsorption and consequent activation of the coagulation system. However, this possible explanation is currently speculative and needs further specific analysis. Nevertheless, this concept is supported by a report stating that the hydroxyl groups located at crystalline surfaces, especially at amorphous areas, are able to react with molecules in their direct neighbourhood [12]. Further, this would be in line with the observation of increased TAT generation on PAD grafts, as dried BNC holds more hydroxyl groups that interact with plasma proteins [31]. Apart, these discrepancies might be also dependent to additional factors like fiber diameter, fiber alignment and the pore size that might contribute to the activation of coagulation system by OIS grafts. However, SAC BNC grafts were comparable to the commercial grafts, PET and ePTFE, indicating the future perspectives of SAC BNC grafts in clinical use.

Next, the interactions between BNC graft materials and the complement cascade system are considered pivotal as complement components could be easily activated upon platelet activation that are adhering to the BNC graft surfaces, where complement components bind to their receptors on the activated platelets. While complement factors such as C1, C3a, factor B and TCC that were secreted by endothelial cells lead to atherosclerosis and vascular occlusion, the blocking of complement activation has proven to subside atherosclerosis and occlusion process [32]. In this study, we have analyzed the complement components C3a and TCC, which are considered to be valid markers for an activated complement system [33, 34]. Consistent with several reports, we have also observed higher plasma levels of complement components in all BNC grafts when compared to the commercial grafts [5, 35, 36]. Though not reaching the level of significance, our study has revealed a decreased tendency for plasma levels of TCC in SAC BNC grafts.

In line with this, there is a study showing that the therapeutic inhibition of TCC, and not C3a, could be more advantageous to minimize the complement induced inflammation [37]. Though the mechanisms of complement component activation by graft material are yet not fully understood, we assume that a chemically active BNC-surface with functional groups (hydroxyl,–OH groups) might activate these complement components [36]. Since all our produced BNC grafts contain many free–OH groups, we did not observe any significant differences between the different BNC grafts. We therefore speculate that the surface chemical entity i.e. reactive–OH groups might serve as the main driver for complement activation and not the surface structure. Therefore, one could improve the compatibility of the BNC grafts to the complement system by reducing or modifying the free-OH groups on the produced cellulose.

Since oxidative stress is an important mediator of atherothrombotic events in cardiovascular disease [38], we have chosen to measure extracellular ROS as a stress representing parameter. ROS are not only produced by activated platelets [39] but also by activated leukocytes that are equally involved in the formation of thrombus [40]. Since our whole blood represents the composite of platelets and leukocytes, the wholesome production of ROS by two major blood cell population could strongly indicate oxidative damage status in the Chandler loop system embedded with different types of grafts. Furthermore, the cfDNA that is released by the blood cells, either due to apoptosis, necrosis, or by neutrophils (as neutrophil extracellular traps), are recognized as an important link between coagulation and inflammation [41]. This circulating cfDNA, which has been shown to be elevated during inflammatory processes [42], further induces ROS production dictating its indirect role in oxidative damage [43]. However, we could not demonstrate any significant changes neither in the level of cfDNA nor in ROS levels for all the analyzed BNC grafts when compared to the commercial grafts. In fact, the ROS levels remained quite low and were comparable between the grafts, indicating that the graft-triggered cellular stress response can be considered to be minimal after 4 hours of blood rotation.

### Conclusions

Based on the data obtained, the lower surface roughness of SAC BNC grafts resulted in less thrombocyte adhesion and activation, less leukocyte adhesion and reduced generation of TAT complex as confirmed by CLSM, SEM, IF, scintigraphy images and ELISA, suggesting that the synthesized SAC structured BNC grafts could be a potential future candidate for anti-thrombogenicity, which is particularly useful for artificial small diameter vascular/blood prostheses. However, the fiber diameter, alignment, pore size along with protein adsorbing properties for SAC BNC grafts need to be thoroughly investigated for their usage in any in vivo settings.

## Supporting information

S1 Table Overall results of blood cell frequencies. The values represented are relative to the baseline cell frequencies that were measured immediately upon blood drawing. SD: Standard deviation.
(DOCX)

S2 Table Measurements and calculations of acquired scintigraphies. MBq: megabequerel
(DOCX)

S3 Table Results of analyzed plasmatic blood components. The values represented are relative to the baseline values that were measured immediately upon blood drawing. SD: Standard deviation.
(DOCX)

S1 Fig Positive and negative controls for immunofluorescence stainings. Negative controls represent stainings without primary antibody. In the first row, red blood cells are stained against CD235a (red) and in the second row, leukocytes are stained against 4′,6-diamidino-2-phenylindole (DAPI, blue) after isolation by ficoll density centrifugation. The cells were given on a small piece of BNC (*), frozen in compound and thereafter sectioned. In the third row, activated platelets are stained against CD62p (green). Platelet rich plasma was produced from whole blood by centrifugation. Thereafter, platelets were activated with adenosine tri phosphate and spread out on a slide. All immunofluorescence images are given with the associated phase contrast image.
(TIF)

## References

[1] Rychlik IJ, Davey P, Murphy J, O'Donnell ME. A meta-analysis to compare Dacron versus polytetrafluroethylene grafts for above-knee femoropopliteal artery bypass. J Vasc Surg. 2014;60(2):506–15. 10.1016/j.jvs.2014.05.049 . 24973288

[2] Hehrlein FW, Schlepper M, Loskot F, Scheld HH, Walter P, Mulch J. The use of expanded polytetrafluoroethylene (PTFE) grafts for myocardial revascularization. J Cardiovasc Surg (Torino). 1984;25(6):549–53. . 6334688

[3] Wang X, Lin P, Yao Q, Chen C. Development of small-diameter vascular grafts. World J Surg. 2007;31(4):682–9. 10.1007/s00268-006-0731-z . 17345123

[4] Pashneh-Tala S, MacNeil S, Claeyssens F. The Tissue-Engineered Vascular Graft-Past, Present, and Future. Tissue Eng Part B Rev. 2015 10.1089/ten.teb.2015.0100 26447530 PMC4753638

[5] Fink H, Hong J, Drotz K, Risberg B, Sanchez J, Sellborn A. An in vitro study of blood compatibility of vascular grafts made of bacterial cellulose in comparison with conventionally-used graft materials. J Biomed Mater Res A. 2011;97(1):52–8. 10.1002/jbm.a.33031 . 21308986

[6] Wippermann J, Schumann D, Klemm D, Kosmehl H, Salehi-Gelani S, Wahlers T. Preliminary results of small arterial substitute performed with a new cylindrical biomaterial composed of bacterial cellulose. Eur J Vasc Endovasc Surg. 2009;37(5):592–6. 10.1016/j.ejvs.2009.01.007 . 19231251

[7] Weber C, Reinhardt S, Eghbalzadeh K, Wacker M, Guschlbauer M, Maul A, et al Patency and in vivo compatibility of bacterial nanocellulose grafts as small-diameter vascular substitute. J Vasc Surg. 2017 10.1016/j.jvs.2017.09.038 . 29248244

[8] Scherner M, Reutter S, Klemm D, Sterner-Kock A, Guschlbauer M, Richter T, et al In vivo application of tissue-engineered blood vessels of bacterial cellulose as small arterial substitutes: proof of concept? J Surg Res. 2014;189(2):340–7. 10.1016/j.jss.2014.02.011 . 24726059

[9] Weber M, Steinle H, Golombek S, Hann L, Schlensak C, Wendel HP, et al Blood-Contacting Biomaterials: In Vitro Evaluation of the Hemocompatibility. Front Bioeng Biotechnol. 2018;6:99 10.3389/fbioe.2018.00099 30062094 PMC6054932

[10] International Organisation for Standardisation. DIN ISO 10993–4: Biological evaluation of medical devices—Part 4: Selection of tests for interactions with blood. 2017.

[11] Klemm D, Marsch S, Schumann D, Udhardt U, inventorsMethod and device for producing shaped microbial cellulose for use as biomaterial, especially for microsurgery. Germany2000.

[12] Klemm D, Cranston ED, Fischer D, Gama M, Kedzior SA, Kralisch D, et al Nanocellulose as a natural source for groundbreaking applications in materials science: Today’s state. Materials Today. 2018;21(7):720–48. 10.1016/j.mattod.2018.02.001.

[13] Klemm D, Kopsch V, Koth D, Kramer F, Moritz S, Richter T, et al., inventors; KKF UG, assignee. Device, Used To Prepare Hollow Bodies Made Of Microbial Cellulose, Includes Template, A First Reservoir, Which Is Filled With A Mixture Comprising A Liquid Culture Medium And A Polymer-forming Microorganism, A Wetting Device, And A Housing. DE patent DE 102012201272 A1. 2013 2012/01/30.

[14] Schindelin J, Arganda-Carreras I, Frise E, Kaynig V, Longair M, Pietzsch T, et al Fiji: an open-source platform for biological-image analysis. Nat Methods. 2012;9(7):676–82. 10.1038/nmeth.2019 22743772 PMC3855844

[15] Golightly M. Dihydrorhodamine (DHR) flow cytometry test for Chronic Granulomatous Disease (CGD): A simple test for rouine clinical flow cytometry2011 27.01.2019; II(1, Winter 2011). Available from: https://www.cytometry.org/newsletters/eICCS-2-1/article6.php.

[16] Rodrigues M, Sinzinger H, Thakur M, Becker W, Dewanjee M, Ezekowitz M, et al Labelling of platelets with indium-111 oxine and technetium-99m hexamethylpropylene amine oxime: suggested methods. International Society of Radiolabelled Blood Elements (ISORBE). Eur J Nucl Med. 1999;26(12):1614–6. 10.1007/s002590050503 . 10638415

[17] Esposito CJ, Popescu WM, Rinder HM, Schwartz JJ, Smith BR, Rinder CS. Increased leukocyte-platelet adhesion in patients with graft occlusion after peripheral vascular surgery. Thromb Haemost. 2003;90(6):1128–34. 10.1160/TH03-04-0226 . 14652647

[18] Milleret V, Hefti T, Hall H, Vogel V, Eberli D. Influence of the fiber diameter and surface roughness of electrospun vascular grafts on blood activation. Acta Biomater. 2012;8(12):4349–56. 10.1016/j.actbio.2012.07.032 . 22842036

[19] Hecker JF, Scandrett LA. Roughness and thrombogenicity of the outer surfaces of intravascular catheters. J Biomed Mater Res. 1985;19(4):381–95. 10.1002/jbm.820190404 . 4055822

[20] Park JY, Gemmell CH, Davies JE. Platelet interactions with titanium: modulation of platelet activity by surface topography. Biomaterials. 2001;22(19):2671–82. 10.1016/s0142-9612(01)00009-6 . 11519787

[21] Kammerer PW, Gabriel M, Al-Nawas B, Scholz T, Kirchmaier CM, Klein MO. Early implant healing: promotion of platelet activation and cytokine release by topographical, chemical and biomimetical titanium surface modifications in vitro. Clin Oral Implants Res. 2012;23(4):504–10. 10.1111/j.1600-0501.2011.02153.x . 21435015

[22] Vogler EA. Protein adsorption in three dimensions. Biomaterials. 2012;33(5):1201–37. 10.1016/j.biomaterials.2011.10.059 22088888 PMC3278642

[23] Falkenback D, Lundberg F, Ribbe E, Ljungh A. Exposure of plasma proteins on Dacron and ePTFE vascular graft material in a perfusion model. Eur J Vasc Endovasc Surg. 2000;19(5):468–75. 10.1053/ejvs.1999.1075 . 10828226

[24] Satyanarayana KG, Rangan A, Prasad VS, Magalhães WLE. Preparation, Characterization, and Applications of Nanomaterials (Cellulose, Lignin, and Silica) from Renewable (Lignocellulosic) Resources. Handbook of Composites from Renewable Materials. 2017:1–66. 10.1002/9781119441632.ch126

[25] Oshima T, Taguchi S, Ohe K, Baba Y. Phosphorylated bacterial cellulose for adsorption of proteins. Carbohydrate Polymers—CARBOHYD POLYM. 2011;83:953–8. 10.1016/j.carbpol.2010.09.005

[26] Lin Q, Zheng Y, Wang G, Shi X, Zhang T, Yu J, et al Protein Adsorption Behaviors of Carboxymethylated Bacterial Cellulose Membranes. International journal of biological macromolecules. 2014;73 10.1016/j.ijbiomac.2014.11.011 25463321

[27] Ogawa R, J. W, I. K. Domain-controlled polymer alloy composed of segmented polyurethane and phospholipid polymer for biomedical applications. Science and Technology of Advanced Materials. 2003;4(6):523–30. 10.1016/j.stam.2003.10.030

[28] Miao S, Shu D, Zhu Y, Lu M, Zhang Q, Pei Y, et al Cancer cell-derived immunoglobulin G activates platelets by binding to platelet FcgammaRIIa. Cell Death Dis. 2019;10(2):87 10.1038/s41419-019-1367-x 30692520 PMC6349849

[29] Yi M, Sakai T, Fassler R, Ruoslahti E. Antiangiogenic proteins require plasma fibronectin or vitronectin for in vivo activity. Proc Natl Acad Sci U S A. 2003;100(20):11435–8. 10.1073/pnas.1635112100 13679585 PMC208775

[30] Osorio M, Canas A, Puerta J, Diaz L, Naranjo T, Ortiz I, et al Ex Vivo and In Vivo Biocompatibility Assessment (Blood and Tissue) of Three-Dimensional Bacterial Nanocellulose Biomaterials for Soft Tissue Implants. Sci Rep. 2019;9(1):10553 10.1038/s41598-019-46918-x 31332259 PMC6646330

[31] Wei Q, Becherer T, Angioletti-Uberti S, Dzubiella J, Wischke C, Neffe AT, et al Protein interactions with polymer coatings and biomaterials. Angew Chem Int Ed Engl. 2014;53(31):8004–31. 10.1002/anie.201400546 . 25045074

[32] Radke D, Jia W, Sharma D, Fena K, Wang G, Goldman J, et al Tissue Engineering at the Blood-Contacting Surface: A Review of Challenges and Strategies in Vascular Graft Development. Adv Healthc Mater. 2018;7(15):e1701461 10.1002/adhm.201701461 29732735 PMC6105365

[33] Gong J, Larsson R, Ekdahl KN, Mollnes TE, Nilsson U, Nilsson B. Tubing loops as a model for cardiopulmonary bypass circuits: both the biomaterial and the blood-gas phase interfaces induce complement activation in an in vitro model. J Clin Immunol. 1996;16(4):222–9. 10.1007/BF01541228 . 8840224

[34] Johnson RJ. Complement activation by biomaterials. Prog Clin Biol Res. 1990;337:507–12. . 2353012

[35] Frank RD, Weber J, Dresbach H, Thelen H, Weiss C, Floege J. Role of contact system activation in hemodialyzer-induced thrombogenicity. Kidney Int. 2001;60(5):1972–81. 10.1046/j.1523-1755.2001.00009.x . 11703617

[36] Leitao AF, Gupta S, Silva JP, Reviakine I, Gama M. Hemocompatibility study of a bacterial cellulose/polyvinyl alcohol nanocomposite. Colloids Surf B Biointerfaces. 2013;111:493–502. 10.1016/j.colsurfb.2013.06.031 . 23880088

[37] Busche MN, Stahl GL. Role of the complement components C5 and C3a in a mouse model of myocardial ischemia and reperfusion injury. Ger Med Sci. 2010;8 10.3205/000109 20930931 PMC2940219

[38] Loscalzo J. Oxidant stress: a key determinant of atherothrombosis. Biochem Soc Trans. 2003;31(Pt 5):1059–61. 10.1042/bst0311059 . 14505479

[39] Qiao J, Arthur JF, Gardiner EE, Andrews RK, Zeng L, Xu K. Regulation of platelet activation and thrombus formation by reactive oxygen species. Redox Biol. 2018;14:126–30. 10.1016/j.redox.2017.08.021 ; PubMed Central PMCID: PMC5596263. 28888895 PMC5596263

[40] Swystun LL, Liaw PC. The role of leukocytes in thrombosis. Blood. 2016;128(6):753–62. 10.1182/blood-2016-05-718114 . 27354721

[41] Gould TJ, Vu TT, Stafford AR, Dwivedi DJ, Kim PY, Fox-Robichaud AE, et al Cell-Free DNA Modulates Clot Structure and Impairs Fibrinolysis in Sepsis. Arterioscler Thromb Vasc Biol. 2015;35(12):2544–53. 10.1161/ATVBAHA.115.306035 . 26494232

[42] Solakoglu O, Steinbach B, Gotz W, Heydecke G, Pantel K, Schwarzenbach H. Characterization of circulating DNA in plasma of patients after allogeneic bone grafting. Clin Oral Investig. 2019;23(12):4243–53. 10.1007/s00784-019-02867-3 . 30826920

[43] Ermakov AV, Konkova MS, Kostyuk SV, Izevskaya VL, Baranova A, Veiko NN. Oxidized extracellular DNA as a stress signal in human cells. Oxid Med Cell Longev. 2013;2013:649747 10.1155/2013/649747 23533696 PMC3606786

