# Supplementary material for: In vitro hemo- and cytocompatibility of bacterial nanocelluose small diameter vascular grafts: Impact of fabrication and surface characteristics
Source: PLoS One. 2020 Jun 24;15(6):e0235168. doi: 10.1371/journal.pone.0235168 (PMC7313737; doi:10.1371/journal.pone.0235168)
Supplement: S1 Table — The values represented are relative to the baseline cell frequencies that were measured immediately upon blood drawing. SD: Standard deviation. (DOCX) [file pone.0235168.s001.docx]

|  | PET | ePTFE | OIS | INV | PAD | SAC | STD | CTRL |
| --- | --- | --- | --- | --- | --- | --- | --- | --- |
| **Erythrocytes** |  |  |  |  |  |  |  |  |
| 120 minutes, mean ± SD | 1.03 ± 0.07 | 1.02 ± 0.03 | 0.97 ± 0.04 | 1.02 ± 0.07 | 0.96 ± 0.05 | 0.99 ± 0.04 | 1 ± 0.07 | 1.02 ± 0.03 |
| 240 minutes, mean ± SD | 0.96 ± 0.08 | 1.01 ± 0.05 | 0.95 ± 0.07 | 0.97 ± 0.05 | 0.95 ± 0.06 | 1.01 ± 0.05 | 0.97 ± 0.08 | 1.01 ± 0.02 |
| **Thrombocytes** |  |  |  |  |  |  |  |  |
| 120 minutes, mean ± SD | 0.81 ± 0.42 | 0.84 ± 0.14 | 0.76 ± 0.19 | 0.48 ± 0.30 | 0.63 ± 0.18 | 0.57 ± 0.35 | 0.63 ± 0.44 | 0.82 ± 0.15 |
| 240 minutes, mean ± SD | 0.31 ± 0.27 | 0.87 ± 0.14 | 0.47 ± 0.42 | 0.23 ± 0.30 | 0.34 ± 0.30 | 0.63 ± 0.23 | 0.38 ± 0.38 | 0.85 ± 0.03 |
| **Leukocytes** |  |  |  |  |  |  |  |  |
| 120 minutes, mean ± SD | 0.92 ± 0.12 | 0.93 ± 0.04 | 0.86 ± 0.09 | 0.84 ± 0.10 | 0.88 ± 0.06 | 0.87 ± 0.08 | 0.91 ± 0.10 | 0.95 ± 0.05 |
| 240 minutes, mean ± SD | 0.75 ± 0.14 | 0.91 ± 0.05 | 0.61 ± 0.35 | 0.51 ± 0.29 | 0.63 ± 0.33 | 0.93 ± 0.03 | 0.53 ± 0.32 | 0.92 ± 0.01 |
| **Neutrophils** |  |  |  |  |  |  |  |  |
| 240 minutes, mean ± SD | 1.13 ± 0.06 | 1.14 ± 0.04 | 1.17 ± 0.03 | 0.84 ± 0.38 | 1.12 ± 0.09 | 1.1 ± 0.11 | 1.2 ± 0.07 | 1.17 ± 0.08 |
| **Lymphocytes** |  |  |  |  |  |  |  |  |
| 240 minutes, mean ± SD | 0.84 ± 0.05 | 0.66 ± 0.06 | 0.65 ± 0.09 | 1.26 ± 0.41 | 0.8 ± 0.20 | 0.76 ± 0.21 | 0.75 ± 0.22 | 0.72 ± 0.12 |
| **Monocytes** |  |  |  |  |  |  |  |  |
| 240 minutes, mean ± SD | 0.97 ± 0.03 | 1.31 ± 0.16 | 1.12 ± 0.19 | 0.62 ± 0.31 | 0.97 ± 0.47 | 1.25 ± 0.27 | 0.81 ± 0.31 | 1.32 ± 0.19 |
