# Supplementary material for: In vitro hemo- and cytocompatibility of bacterial nanocelluose small diameter vascular grafts: Impact of fabrication and surface characteristics
Source: PLoS One. 2020 Jun 24;15(6):e0235168. doi: 10.1371/journal.pone.0235168 (PMC7313737; doi:10.1371/journal.pone.0235168)
Supplement: S2 Table — MBq: megabequerel (DOCX) [file pone.0235168.s002.docx]

|  | RawIntdent | calculated radioactivity (MBq) | calculated number of thrombocytes |
| --- | --- | --- | --- |
| Reference | 270812 | 5.313 | - |
| Control loop without rotation | 1194 | 0.023 | 1192536 |
| Control loop with rotation | 2222 | 0.044 | 2219276 |
| PET | 40849 | 0.801 | 40798921 |
| ePTFE | 1689 | 0.033 | 1686929 |
| OIS | 36651 | 0.719 | 36606068 |
| INV | 25559 | 0.501 | 25527666 |
| PAD | 42043 | 0.825 | 41991458 |
| SAC | 13953 | 0.274 | 13935894 |
| STD | 34188 | 0.671 | 64146087 |
