# Supplementary material for: In vitro hemo- and cytocompatibility of bacterial nanocelluose small diameter vascular grafts: Impact of fabrication and surface characteristics
Source: PLoS One. 2020 Jun 24;15(6):e0235168. doi: 10.1371/journal.pone.0235168 (PMC7313737; doi:10.1371/journal.pone.0235168)
Supplement: S3 Table — The values represented are relative to the baseline values that were measured immediately upon blood drawing. SD: Standard deviation. (DOCX) [file pone.0235168.s003.docx]

|  | PET | ePTFE | OIS | INV | PAD | SAC | STD | CTRL |
| --- | --- | --- | --- | --- | --- | --- | --- | --- |
| **ROS** |  |  |  |  |  |  |  |  |
| 240 minutes, mean ± SD | 1.40 ± 0.14 | 0.43 ± 0.25 | 1.50 ± 0.79 | 0.76 ± 0.31 | 1.60 ± 1.70 | 1.00 ± 0.16 | 0.93 ± 0.26 | 0.82 ± 0.39 |
| **cfDNA** |  |  |  |  |  |  |  |  |
| 240 minutes, mean ± SD | 2.8 ± 3.10 | 8.08 ± 4.34 | 9.82 ± 5.49 | 4.49 ± 3.16 | 7.14 ± 7.10 | 8.01 ± 5.10 | 10.67 ± 8.21 | 6.40 ± 2.60 |
| **TCC** |  |  |  |  |  |  |  |  |
| 240 minutes, mean ± SD | 7.59 ± 3.27 | 8.85 ± 2.76 | 23.31 ± 23.01 | 41.08 ± 35.23 | 39.08 ± 42.79 | 28.99 ± 20.28 | 45.89 ± 46.62 | 8.06 ± 0.82 |
| **TAT** |  |  |  |  |  |  |  |  |
| 240 minutes, mean ± SD | 1.59 ± 0.57 | 1.78 ± 0.36 | 2.88 ± 1.76 | 1.63 ± 1.05 | 1.73 ± 0.78 | 1.20 ± 0.18 | 4.57 ± 3.08 | 1.97 ± 0.25 |
| **C3a** |  |  |  |  |  |  |  |  |
| 240 minutes, mean ± SD | 16.20 ± 12.19 | 15.65 ± 10.24 | 41.81 ± 44.07 | 39.88 ± 40.63 | 54.48 ± 46.11 | 60.69 ± 53.15 | 52.24 ± 59.06 | 20.61 ± 6.66 |
