# Supplementary figures and images for: In vitro hemo- and cytocompatibility of bacterial nanocelluose small diameter vascular grafts: Impact of fabrication and surface characteristics
Source: PLoS One. 2020 Jun 24;15(6):e0235168. doi: 10.1371/journal.pone.0235168 (PMC7313737; doi:10.1371/journal.pone.0235168)

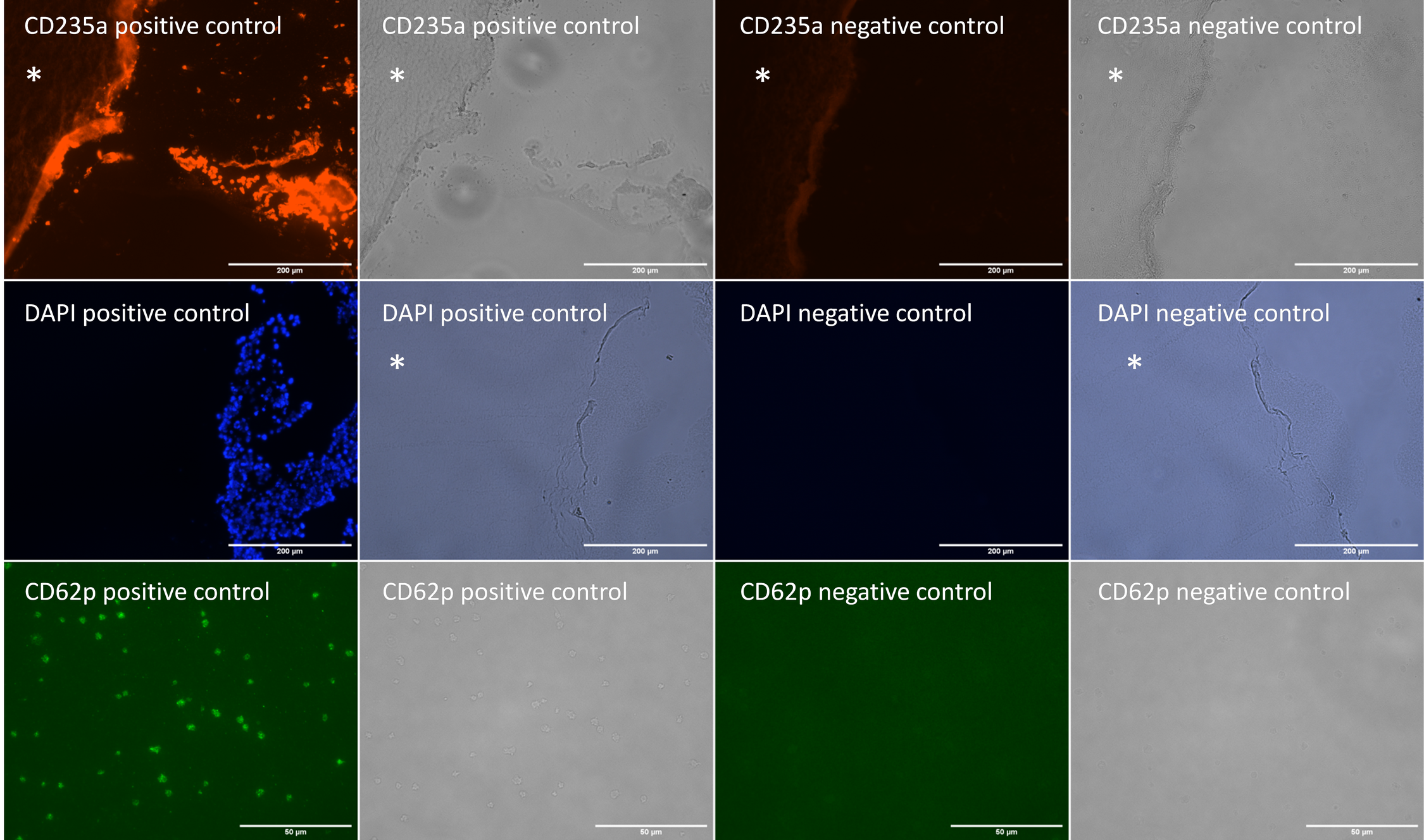

Supplement: S1 Fig — Negative controls represent stainings without primary antibody. In the first row, red blood cells are stained against CD235a (red) and in the second row, leukocytes are stained against 4′,6-diamidino-2-phenylindole (DAPI, blue) after isolation by ficoll density centrifugation. The cells were given on a small piece of BNC (*), frozen in compound and thereafter sectioned. In the third row, activated platelets are stained against CD62p (green). Platelet rich plasma was produced from whole blood by centrifugation. Thereafter, platelets were activated with adenosine tri phosphate and spread out on a slide. All immunofluorescence images are given with the associated phase contrast image. (TIF) [file pone.0235168.s004.tif]
